# Profiles of the auditory epithelia related microRNA expression in neonatal and adult rats

**DOI:** 10.1186/s40001-014-0048-6

**Published:** 2014-09-06

**Authors:** Weiwei Guo, Yongyi Yuan, Zhaohui Hou, Xiang Wang, Shiming Yang

**Affiliations:** Department of Otolaryngology, Head & Neck Surgery, Institute of Otolaryngology, Chinese PLA General Hospital, 28# Fuxing Road, Beijing, 100853 China

**Keywords:** miRNA, Microarray, Neonatal, Adult, Rat, Cochlear

## Abstract

**Background:**

The impact of miRNA differential expression on the auditory epithelium stem cell development in postnatal rats is not clear. The present study was designed to analyze miRNA expression in the organ of Corti of neonatal and adult rats.

**Methods:**

The cochleae of newborn (P0) and adult (P30) Sprague-Dawley rats were dissected in cold PBS to collect the sensory epithelia. Small RNAs were extracted using the mirVana RNA Isolation kit. Then, miRNA expression profiling was performed with RNAs from three newborns and three adult rats utilizing the TaqMan Array Rodent MicroRNA Panel.

**Results:**

Eighteen miRNAs were found be differentially expressed, 16 were unregulated in mature cochleae with the fold changes ranging from 17 to 600 folds. The expression levels of two miRNAs were reduced in the mature rat cochleae. GO analysis and signaling pathway analysis revealed the potential involvement of the miRNAs in the regulation of Wnt and TGF-β signaling pathways in hair cell development.

**Conclusions:**

Our results provided novel insights into the functional significance of miRNAs in the basilar membrane cells development, and revealed the potential importance of miRNAs in the hair cell by regulation of Wnt and TGF-β signaling.

## Background

The inner ear, located in the temporal bone, contains the cochlea responsible for hearing and the vestibule responsible for balance. In the cochlea, the organ of Corti is a specialized structure that responds to fluid-borne vibrations. The organ of Corti is a complex organ that comprises a highly-ordered cellular mosaic of sensory hair cells (HCs) and non-sensory supporting cells (SCs). The generation of new HCs occurs throughout life in the auditory and vestibular sensory receptor of fishes and amphibians [1,2]. In mammals, embryonic HC and SC proliferation within the sensory epithelium culminates between embryonic day 13 (E13) and E15, but stops after birth [3]. It has been shown that acutely dissociated cells from the newborn rat or young rat organ of Corti can develop into otospheres consisting of 98% nestin cells when plated on a non-adherent substratum in the presence of either epidermal growth factors or fibroblast growth factors [4,5]. Li and colleagues have shown that the adult utricular sensory epithelium contains cells that display the biological features of stem cells including self-renewal, sphere formation and capability of differentiating into hair cell-like cells [6]. However, the replacement of lost hair cells does not occur spontaneously in pathological conditions such as age-related cochlear degeneration. A number of genes that affect various aspects of inner ear development have been identified and include transcription factors, morphogens, growth factors, receptors, and so on.

microRNAs (miRNAs) were discovered by Lee and colleague in *Caenorhabditis elegans* in 1993 as novel molecules that play an important role in gene expression regulation [7]. miRNAs are small noncoding RNA molecules (approximately 22 nucleotides) that regulate posttranscriptional gene expression by relatively nonspecific binding to the 3’-untranslated region of mRNA [8]. A single miRNA may regulate several genes because of the sequence similarity. It has been proposed that over one third of all protein-encoding genes are under translational control by miRNA [9]. miRNAs are involved in a variety of cellular processes, including cellular differentiation, proliferation and apoptosis [10]. miRNAs play an essential role in inner ear development [11]. A recent study using conditionally knocked out Dicer only in the inner ear, SE hair and SCs after their normal differentiation from progenitor cells revealed the importance of miRNAs in inner ear development and function in vertebrates [12]. Using an *in silico* prediction model that integrates miRNAs, mRNA and protein expression, Elkan-Miller and co-workers discovered the expression of 157 miRNAs in the inner ear sensory epithelium, with 53 miRNAs differentially expressed between the cochlea and the vestibule. Six miRNA families appear to be functionally important in the inner ear [13].

Zhang and colleagues [14] identified the miRNAs involved in degeneration of the organ of Corti during age-related hearing loss. They showed that 111 and 71 miRNAs exhibited differential expression in the C57 and CBA mice aged from postnatal day 21 to 16 months, respectively, and that downregulated miRNAs substantially outnumbered upregulated miRNAs during aging. However, comparisons of miRNA differential expression in the organ of Corti between newborn and adult rats, representing the early development of the inner ear sensory epithelium, have not yet been investigated. Therefore, in this study, we characterized the miRNAs expression profile in the auditory epithelia in both newborn and adult rats in order to examine the patterns and potential roles of miRNA differential expression in the early development of the inner ear sensory epithelium. The results showed that 16 differentially expressed miRNAs were identified. GO (Gene Ontology) term analysis revealed the importance of Wnt and transforming growth factor (TGF)-β signaling in the hair cell development. Understanding the miRNA and gene interaction network shed light on their roles on the development of normal and impaired hearing, and the mechanisms leading towards deafness.

## Methods

### Animal

Neonatal (P0) and adult (P30) Sprague-Dawley (SD) rats were approved by the Institutional Animal Care and Use Committees of Chinese PLA General Hospital.

### RNA isolation

The cochleae of new born (P0) and adult (P30) SD rats were dissected in cold PBS (10 mM Na_2_HPO_4_,_,_1.7 mM KH_2_PO_4_,137 mM NaCl, 2.7 mM KCl, pH 7.4) to collect the sensory epithelia. The collected tissues were stored in RNAlater (Ambion, Austin, TX, USA) until use. Small RNAs (<200 nucleotides) were extracted using the mirVana RNA Isolation kit (Ambion, Austin, TX, USA) according to the manufacture’s instruction. The quality and quantity of the RNA preparations were determined using a 2100 Agilent BioAnalyzer and a NanoDrop ND-1000 spectrophotometer (Thermo Scientific, Wilmington, DE, USA).

### Microarray analyses

miRNA expression profiling was performed with RNAs from three newborns and three adult rats utilizing the TaqMan Array Rodent MicroRNA Panel (Applied Biosystems, Foster City, CA, USA) using 50 ng of RNA per port for a total of 400 ng. This array contains 365 miRNA targets as well as endogenous controls. Normalization was performed with the small nuclear RNAs (snRNAs) U44 and U48. These snRNAs are stably-expressed reference genes suitable for normalization of miRNAs. The qRT-PCR for the assessment of gene expression levels was performed using an ABI Prism 7900HT Sequence detection system (Applied Biosystems, Foster City, CA, USA).

### Two class differential

We applied the random variance model (RVM) *t*-test to filter the differentially expressed miRNAs for the newborn and adult groups because the RVM *t*-test can raise degrees of freedom effectively in the cases of small samples. After the significant analysis and FDR analysis, we selected the differentially expressed genes according to the *P*-value threshold [15].

### GO analysis

GO analysis was applied to analyze the main function of the differential expression genes according to the Gene Ontology which is the key functional classification of National Center for Biotechnology Information (NCBI) [16]. Generally, Fisher’s exact test and *χ*^2^ test were used to classify the GO category, and the false discovery rate (FDR) [17] was calculated to correct the *P*-value, the smaller the FDR, the smaller the error in judging the *P*-value. The FDR was defined as:

$$ FDR=1-\frac{N_k}{T} $$

where *N*_*k*_ refers to the number of Fisher’s test *P*-values less than *χ*^2^ test *P-*values. We computed the *P*-values for the GOs of all the differentially expressed genes. This enrichment analysis provides a measure of the significance of the function: as the enrichment increases, the corresponding function is more specific, which enabled us to identify those GOs with more concrete function description in the experiment. Within the significant category, the enrichment Re was given by:

$$ \mathrm{Re}=\left({n}_f/n\right)/\left({N}_f/N\right) $$

where *n*_*f*_ is the number of differential genes within the particular category, *n* is the total number of genes within the same category, *N*_*f*_ is the number of differential genes in the entire microarray, and *N* is the total number of genes in the microarray [18].

### Pathway analysis

Pathway analysis was used to identify the significant pathway of the differentially expressed genes according to the Kyoto Encyclopedia of Genes and Genomes (KEGG), Biocarta and Reatome. Again, we used the Fisher’s exact test and *χ*^2^ test to select significant pathways. The threshold of significance was defined by *P*-value and FDR. The enrichment Re was calculated using the equation described above [19-21]. The relationship of the miRNAs and genes was counted by their differential expression values, and according to the interactions of miRNA and genes in Sanger MicroRNA database to build the MicroRNA-Gene-Network. The adjacency matrix of MicroRNA and genes A = [ai,j] is made by the attribute relationships among genes and MicroRNA, and [ai,j] represents the relation between the weight of gene i and MicroRNA j. In the MicroRNA-Gene-Network, circles represent genes and squares represent miRNAs, and their relationship is represented by one edge. The center of the network is represented by degree. Degree means the contribution made by one miRNA to the genes around or the contribution made by one gene to the miRNAs around. The key miRNAs and genes in the network always have the largest degrees [22,23].

### MicroRNA-GO-network

The miRNA-GO-network is built according to the relationship between significant GOs and genes and the relationships among miRNAs and genes. The adjacency matrix of MicroRNA and genes A = [ai, j] is made by the attribute relationships among GOs and miRNAs, and [ai,j] represents the relation between the weight of GO i and MicroRNA j. In the MicroRNA-Gene-Network, circles represent genes and squares represent miRNAs, and their relationship is represented by one edge. The center of the network is represented by degree. Degree means the contribution one miRNA to the GOs around or the contribution one GO to the MicroRNAs around. The key miRNA and gene in the network always have the largest degrees.

## Results

### miRNA expression profile analysis

To gain insights into the role of miRNAs that may be associated with the proliferative ability of cochlear cells during maturation of the cochlea, we examined the global expression pattern of mature miRNA using TaqMan® Rodent MicroRNA Arrays V2.0 (Applied Biosystems, Foster City, CA, USA). A total of 18 miRNAs exhibited expression changes in adult cochleae (Table 1) when compared with the newborn cochleae. Among these miRNAs, 16 were unregulated in mature cochleae with the fold changes ranging from 17 to 600 folds. The expression levels of two miRNAs (rno-miR-29c and rno-miR-29a) were reduced in the mature rat cochleae. This observation suggests miRNAs are involved in development process of cochleae.

**Table 1:** **Differential basilar membrane miRNA (microRNA) expression of mature to newborn rat**

**miRNA ID**	**Fold change(2** ^**-△△CT**^ **)**	**Style**	**Sequence**
rno-miR-296-star	601.8687077	up	AGGGCCCCCCCUCAAUCCUGU
rno-miR-183	311.2671462	up	UAUGGCACUGGUAGAAUUCACU
rno-miR-130b	73.88701805	up	CAGUGCAAUGAUGAAAGGGCAU
rno-miR-298	73.54126053	up	GGCAGAGGAGGGCUGUUCUUCCC
rno-miR-199a-5p	39.0786033	up	CCCAGUGUUCAGACUACCUGUUC
rno-miR-106b-star	38.46729557	up	CCGCACUGUGGGUACUUGCUGC
rno-miR-323	37.0342188	up	CACAUUACACGGUCGACCUCU
rno-miR-301b	36.10785966	up	CAGUGCAAUGGUAUUGUCAAAGC
rno-miR-342-5p	19.28817847	up	AGGGGUGCUAUCUGUGAUUGAG
rno-miR-19a	18.83546833	up	UGUGCAAAUCUAUGCAAAACUGA
rno-miR-18a	18.76863728	up	UAAGGUGCAUCUAGUGCAGAUAG
rno-miR-487b	18.75103727	up	AAUCGUACAGGGUCAUCCACUU
rno-miR-20a	18.57354895	up	UAAAGUGCUUAUAGUGCAGGUAG
rno-miR-344a-3p	18.35504823	up	UGAUCUAGCCAAAGCCUGACCGU
rno-miR-431	17.97658408	up	UGUCUUGCAGGCCGUCAUGCA
rno-miR-301a	17.94604464	up	CAGUGCAAUAGUAUUGUCAAAGC
rno-miR-29c	0.071923249	down	UAGCACCAUUUGAAAUCGGUUA
rno-miR-29a	0.035852454	down	UAGCACCAUCUGAAAUCGGUUA

### Microarray-based GO analysis

**Figure 1:**
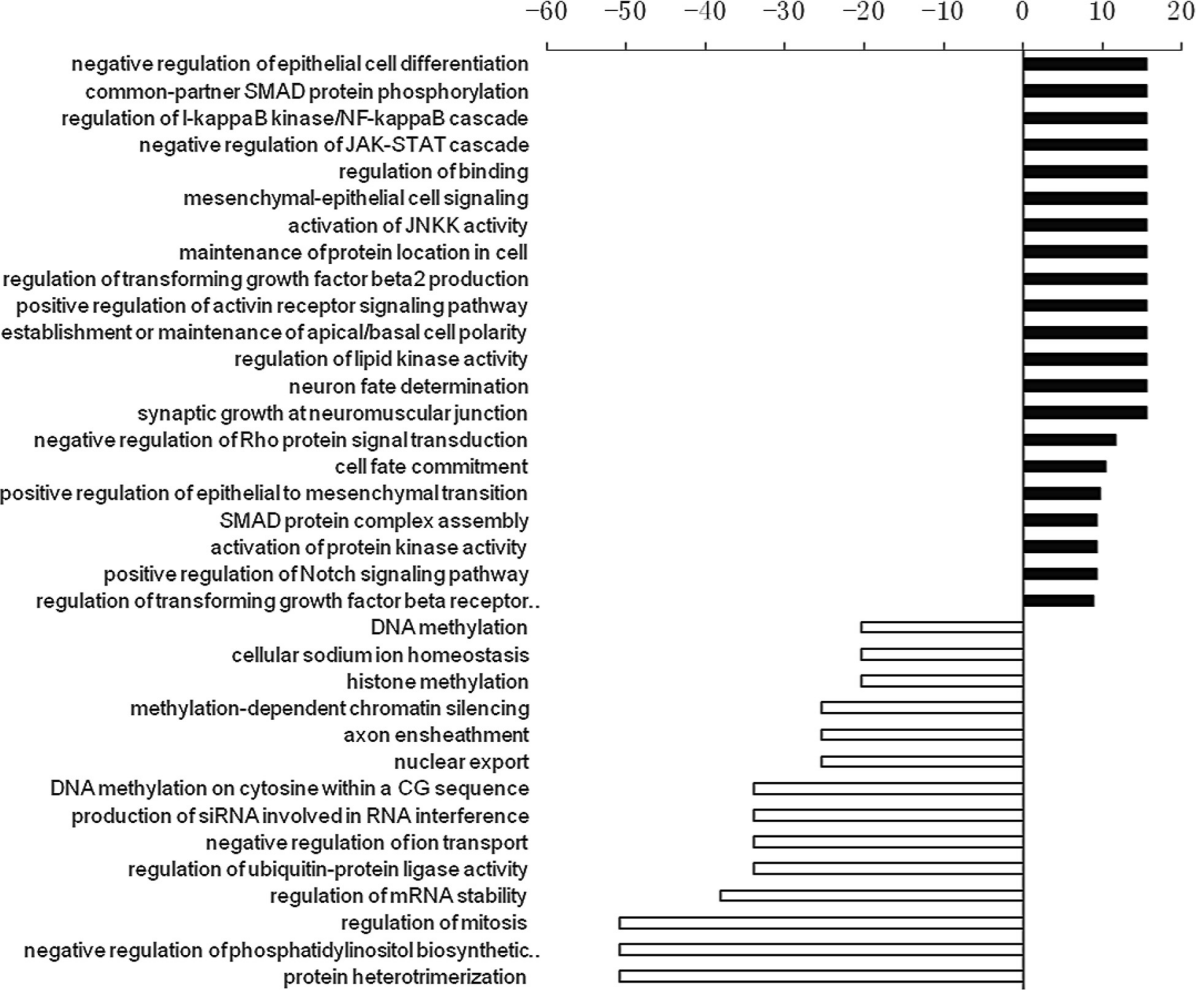
**The GO (Gene Ontology) analysis of the differentially expressed microRNAs (miRNAs).** The solid bars compose the GOs targeted by overexpressed miRNA, and the boxed bar composes the GOs targeted by down-expressed miRNA. All these GOs show increased enrichment. The vertical axis is the GO category and the horizontal axis is the enrichment of GO.

**Figure 2:**
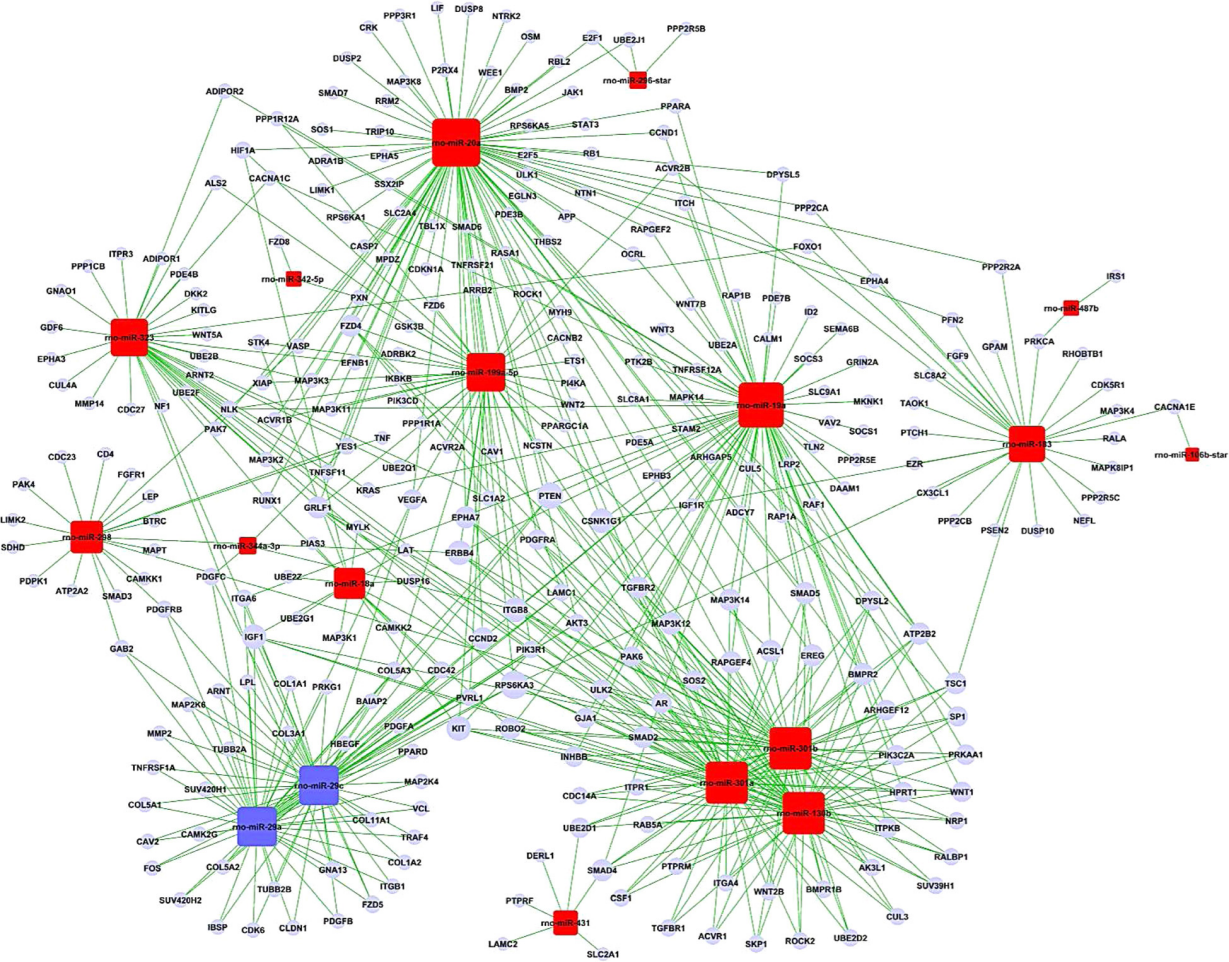
**miRNAs-Gene-Network.** Red box represents unregulated miRNA and blue box downregulated miRNA (microRNA). Edges describe the inhibitive effect of miRNA on mRNA.

**Figure 3:**
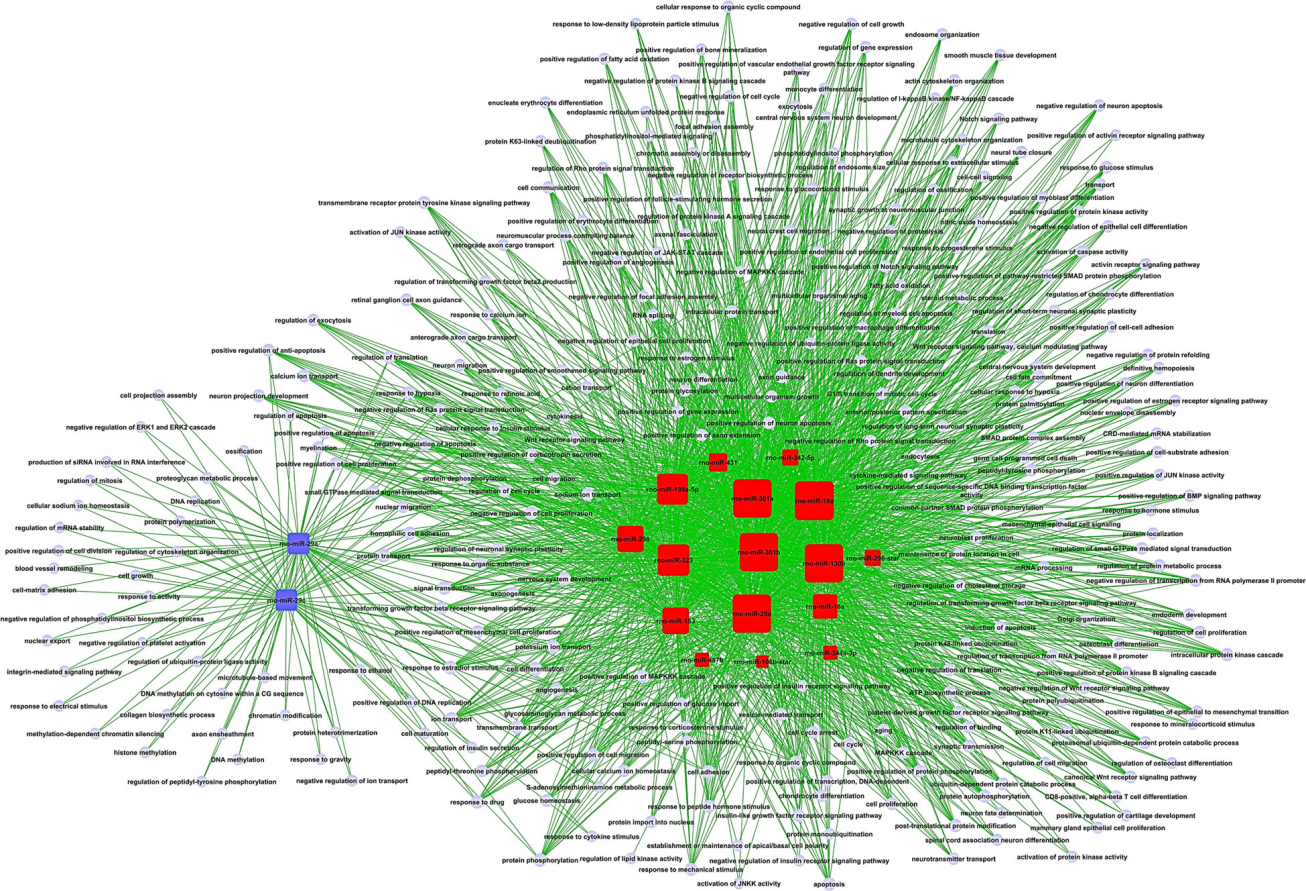
**GO (Gene Ontology) network.** Red box represents unregulated microRNA (miRNA) and blue box downregulated miRNA. Edges describe the inhibitive effect of microRNA on GO.

According to the threshold of GOs significantly regulated by miRNAs, the *P*-value and FDR was < 0.001 and < 0.05, respectively. The high-enrichment GOs targeted by over-expressed miRNAs included negative regulation of epithelial cell differentiation, common-partner SMAD protein phosphorylation, mesenchymal-epithelial cell signaling, regulation of TGF-β2 (Figure 1). In contrast, significant GOs corresponding to downregulated miRNAs included protein heterotrimerization, negative regulation of phosphatidylinositol biosynthetic process and regulation of mitosis. Among these cellular processes, the maximum-enriched-GO relating to TGF-β2 and SMAD signaling suggested that they might have an important role in the proliferation potient of SE. Additionally, the miRNA-mRNA network analysis integrated these miRNAs and GOs by outlining the interactions of miRNA and GO-related genes (Figure 2). The miRNA-mRNA regulatory networks were established (Figure 3) and distinguished the putative target mRNAs between overexpressed and under-expressed miRNAs. Seven overexpressed miRNAs (miR-20a, miR-199a-5p, miR-199, miR-323, miR-301a, miR-301b and miR-130b) showed the most target mRNAs. The miRNAs including miR-301a, miR-301b and miR-130b regulated some important genes, including TGF-βR1, TGF-βR2, Smad2, Smad4 and Smad5 and, therefore, might be of great importance to the activation of the organ of Corti.

### Signaling pathways regulated by differentially expressed miRNAs

**Figure 4:**
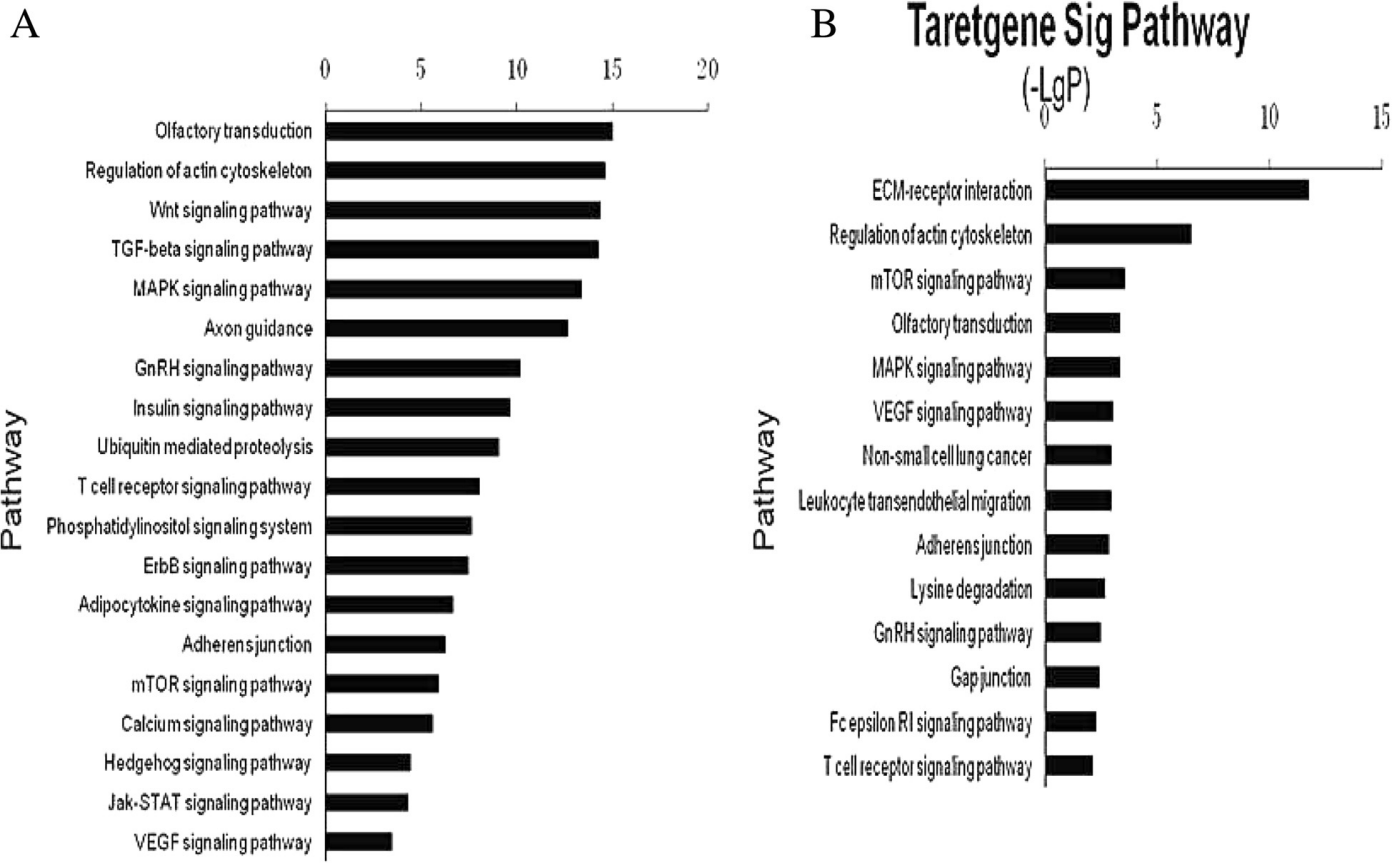
**Pathway analysis based on microRNA (miRNA) targeted genes. (A)** and **(B)** show significant pathways targeted by upregulated and downregulated miRNA, respectively. The vertical axis is the pathway category, and the horizontal axis is the enrichment of pathways. **(A)** Pathways probably upregulated by enrichment of miRNAs in newborn rat; **(B)** Pathways probably downregulated by enrichment of miRNAs in newborn rat.

Functional analysis of miRNAs by KEGG revealed that 19 signal transduction pathways were upregulated (Figure 4A) and 14 were downregulated (Figure 4B). The upregulated signaling pathways including Wnt, TGF-β and mitogen-activated protein kinase (MAPK) have been shown to participate in the activation of stem cells. A wide variety of cellular processes, including regulation of actin cytoskeleton, MAPK and gonadotropin releasing hormone (GnRH) signaling pathway, also featured the functions of significant signaling pathways.

## Discussion

miRNAs have become an area of intense study because of their involvement in human diseases. The lack of inner ear hair cell proliferation contributes to hearing loss in the aging population. In the present study, we compared the miRNA expression profiles between the newborn and adult cochlear sensory epithelia. Our results revealed that several miRNAs were differentially expressed in these two cochleae age groups. The difference in miRNA expression may contribute to the loss of proliferation of sensory cells in the organ of Corti in adult cochleae. These results provide novel insights into the functional significance of miRNAs in the basilar membrane cells development.

Given that embryonic HC and SC proliferation within the sensory epithelia culminates between embryonic day 13 (E13) and E15, but stops after birth in mammalian ears, the fundamental mechanisms behind the embryonic HC and SC proliferation potential are probably lost after birth. One of the possibilities is that coordinated and tightly controlled gene expression programs orchestrate the developmental process. miRNAs as key regulators might play important roles during this phenotypic transition, further adding another layer of complexity to the regulatory network for basilar membrane proliferation. Our miRNAs microarray data suggested that the expression profile of miRNAs in the rat inner ear appears to be well established by P0, consistent with the fact that early inner ear development and cell fate specification mostly occur embryonically [24]. Our profiling data identified two distinct expression patterns of miRNAs between newborn and adult rat basilar membrane. Such differences appeared to be associated with basilar membrane proliferation. The miRNAs expression profiling identified 18 differentially expressed miRNAs with 16 miRNAs being increased and two miRNAs decreased in the mature rat compared to that of the newborn rat. miRNAs that increased mostly in the adult basilar membrane includes miR-296, mi-130b and miR-183 and that those that decreased mostly include miR-29c and miR-29a. MiR-296 has been demonstrated to modulate the pluripotency of embryonic stem cells (ESCs) by repressing the expression of Oct4, Sox2, and Nanog [25]. In vertebrates, the expression domain of conserved miRNA-183 (miR-183) family members appears to be restricted to ciliated neurosensory epithelial cells and certain cranial and spinal ganglia [26,27]. In zebrafish the miRNAs are detected in the eye, nasal epithelium, and sensory hair cells of the ear and neuromasts [26], and injection of miR-183 and miR-200 family members into zebrafish embryos have been demonstrated to impact development and affect neuromast migration [28]. Additionally, expression of miR-183 family members in mouse eye and aural sensory hair cells of the ear has been previously demonstrated [29].

Compared with the differential expression pattern of miRNAs between newborn (P0) and adult rats (P30), Zhang and colleagues [14] reported different or even opposite patterns when they questioned which miRNAs are involved in age-related (from P21 to 16 months) degeneration of the organ of Corti, the auditory sensory epithelium that transduces mechanical stimuli to electrical activity in the inner ear. They showed that 111 and 71 miRNAs exhibited differential expression in the C57 and CBA mice, respectively, and that downregulated miRNAs substantially outnumbered upregulated miRNAs during aging. miRNAs that had approximately 2-fold upregulation included members of miR-29 family and miR-34 family and that were downregulated by about 2-fold were members of the miR-181 family and miR-183 family. The inconsistency between Zhang’s report and our study suggested that miRNA patterns in the organ of Corti change with aging and that miRNAs such as miR-183 and miR29 play different roles in the development of organ of Corti in newborn, younger and older animals.

The present GO analysis and signaling pathway analysis showed that the high-enrichment GOs targeted by over-expressed miRNAs in young adult (P30) compared with newborn rats (P0) included negative regulation of epithelial cell differentiation, common-partner SMAD protein phosphorylation, mesenchymal-epithelial cell signaling, regulation of TGF-β2 production. In contrast, significant GOs corresponding to downregulated miRNAs included protein heterotrimerization, negative regulation of phosphatidylinositol biosynthetic process and regulation of mitosis. Among these cellular processes, the maximum-enriched-GO relating to TGF-β2 and SMAD signaling suggested that they have an important role in the proliferation potient of SE. However, Zhang and colleagues [14] reported that miRNAs upregulated in age-related mice (from P21 to 16 months) are known regulators of pro-apoptotic pathways. In contrast, downregulated miRNAs are known to be important for proliferation and differentiation, respectively. The author concluded that the shift of miRNA expression favoring apoptosis occurred earlier than detectable hearing threshold elevation and hair cell loss. The authors suggested that changes in miRNA expression precede morphological and functional changes, and that upregulation of pro-apoptotic miRNAs and downregulation of miRNAs promoting proliferation and differentiation are both involved in age-related degeneration of the organ of Corti. The inconsistency in functions between Zhang’s report and our study can be explained by the different roles of miRNAs in the development of the organ of Corti in newborn, younger and older animals.

Establishment of primitive streak cells upon differentiation of ESCs depends on the presence of active Wnt and TGF-β/nodal/activin signaling, which recapitulates early events that led to germ-layer induction in the mammalian embryo [30]. In our study, we found that miRNAs that inhibit Wnt and TGF-β signaling pathway were decreased in adult rats. Recently, Oshima and colleagues generated mechanosensitive sensory hair cell-like cells from embryonic and induced pluripotent stem cells by the combination of Wnt inhibitor Dkk1, selective inhibitor of Smad3 (SIS3) which interferes with TGF-β signaling, and insulin-like growth factor 1 (IGF-1) [31]. Consistent with previous studies, our findings revealed the potential importance of miRNAs in the hair cell by regulation of Wnt and TGF-β signaling.

## Conclusions

Our results provided novel insights into the functional significance of miRNAs in the basilar membrane cells development, and revealed the potential importance of miRNAs in the hair cell by regulation of Wnt and TGF-β signaling.

## Abbreviations

ESCs: embryonic stem cells
FDR: false discovery rate
GO: Gene Ontology
HCs: sensory hair cells
IGF-1: insulin-like growth factor 1
KEGG: Kyoto Encyclopedia of Genes and Genomes
MAPK: mitogen-activated protein kinase
GnRH: gonadotropin releasing hormone
miRNAs: microRNAs
NCBI: National Center for Biotechnology Information
PBS: phosphate-buffered saline
PCR: polymerase chain reaction
SCs: non-sensory supporting cells
SD: Sprague-Dawley
snRNAs: small nuclear RNAs
TGF: transforming growth factor
